# Life as an early career researcher: interview with Minhaj Sirajuddin

**DOI:** 10.4155/fsoa-2017-0106

**Published:** 2017-11-01

**Authors:** Minhaj Sirajuddin

**Affiliations:** 1 Center for Cardiovascular Biology and Diseases, Institute for Stem Cell Biology and Regenerative Medicine, GKVK Campus, Bellary Road, Bangalore-560065, Karnataka, India

**Keywords:** cardiomyopathy, cytoskeleton, funding, mentorship and early career scientist challenges

## Abstract

**Minhaj Sirajuddin talks to Francesca Lake, Head of Open Access Publishing.** Minhaj Sirajuddin is currently an Assistant Investigator at the Institute for Stem Cell Biology and Regenerative Medicine (InStem), Bangalore, India. His lab works on understanding biological motility mediated by cytoskeleton elements using biophysics and cell biology. Previously his tenures were at UCSF (CA, USA) for his postdoctoral work and the Max Planck Institute (Dortmund, Germany) for his PhD. Minhaj is a recipient of Wellcome Trust – DBT India Alliance Intermediate Fellowship and the EMBO Young Investigator Award. He was also a finalist in the inaugural Future Science Early Career Research Award.

## Can you tell us a little about your career path to-date?

My first research experience was in the Molecular Biophysics Unit, IISc Bangalore (India) with Raghavan Varadarajan. That was followed by doctoral studies in structural biology with Alfred Wittinghofer at Max Planck Institute for molecular physiology. After that I moved to San Francisco to join Ron Vale's lab at the University of California, San Francisco, to study molecular motors. In November 2014, I moved back to India and completed my cycle and joined as an independent investigator at Institute for Stem Cell Biology and Regenerative Medicine (InStem) located in Bangalore. So, it is been a fairly linear (or circular) path with no major twists and turns. However, I have always been fortunate to be associated with great mentors and colleagues throughout my career, from whom I have learned a lot.

## What are you working on at the moment?

Currently, our lab is interested in understanding the mechanism of molecular machines that mediate biological motility, which includes muscle contraction, motility of cells and microscopic materials inside cells. A major focus is to study cardiomyopathies, a heart muscle disease caused by genetic mutations in protein molecules that perform muscle contraction (heart beat). Our goal is to understand how minute changes in amino acid composition of proteins can manifest full-blown changes in heart morphology and physiology, which potentially could open new therapeutic windows.

## What do you find most rewarding about your work?

The exhilarating feeling of discovering or finding something new and sharing with your colleagues! What's sad, however, is the progressive decay of this exhilaration by the time the work is published. Lately, I am also getting hooked on listening to lab researchers’, excitement in describing their findings – especially in lab meetings, which gives a sense of satisfaction about training young people.

## And what do you find the most challenging?

I think there are three challenges that I am facing, which most of the early career scientists out there might agree on.

First, back to bench, while it is great to see people in your lab getting trained and producing good work, not being in bench is a liability, especially in India where the new Principal Investigator is the lab technician, the senior postdoc and the administrator all rolled in to one. It is a constant battle to beat the forces that keep you out of the lab. The key is inventing new ways to overcome the inertia and finding your way back to the bench.

Second, connecting outside the scientific community, an important component to justify basic research funding. This is perhaps the most challenging, as most of us have zero experience. I am yet to figure or rather adapt an outlet to overcome this challenge.

Finally, being trained by baby boomers and training millennials, I feel the current lot of early career scientists are the fulcrum of change. Most of us have trained in conventional academia settings, be it getting your research funded or disseminating your findings we are still using and even worse training people with this 20th century model, which will become obsolete in future. The challenge is that we have not been trained to tap into new avenues of funding that does not rely on traditional funding bodies. The publishing landscape is also rapidly changing – preprints, double blind and open peer review are something many of us have not experienced and sometimes find uncomfortable too. Similarly, training the workforce in your lab needs to change, not everyone is going to end up in academia. When people join the lab, it is important to make it clear that the linear path of PhD to postdoc to independent job in academia is remote and encouraging then to find alternate paths after PhD.

## In your application to the Future Science Early Career Research Award, you discussed your unconventional approach to (successfully) answer your research questions & your hope to address the gap in genetic research in South Asian populations. What challenges can a researcher face in performing ‘unconventional’ research?

The challenge lies in people not being willing to move outside their comfort zone. It is understandable that one spends years and years on gaining an expertise and wanting to take full advantage, but over the years, it becomes a disadvantage.

## How would you recommend people overcome this?

There are many mechanisms that are already in place, for example, sabbaticals and some senior scientists take it to full advantage. For early career scientists, it can be a bottom-up approach, where one actively engages with a quarter of their time in setting up something new – this is where being in bench helps (as I mentioned earlier). Another thing that helps is surrounding yourself with people tackling biology with diverse ideologies. For me, it is the biggest advantage of my campus (InStem/NCBS/CCAMP). It simply has changed the way I think about biology problems. Another way is top-down, where the hiring committees encourage moving in a new direction.

## One of the biggest challenges facing early career researchers is launching their career with impactful research. Aside from the common adage of publishing in high impact journals, what advice would you give to someone aspiring to do this?

Find a mentor who will care about your career. Often I see people wanting to work on a hot field or to learn the hottest technique in town, but hardly put emphasis on the lab environment or mentor. Just imagine, it this way: the hottest techniques or fields of research might get outdated, but the experience you gain and lessons learned from working alongside amazing colleagues and mentors is everlasting. When choosing your mentor, a quick check will help in understanding the lab environment, for example, ratio of lab people vs stories (papers) coming out, which is a factor often overlooked, alumni listings and talking to current and former members of lab, etc.

## Another challenge is attaining funding. Is this something you have come across, & do you think this is something that is likely to change in the future?

So far so good, but I do expect that to change in the future. Increasingly we are seeing that the funding scope is being shrunken and the taxpayers are demanding accountability of basic science funding. That is why connecting to the nonscientific community is important (as discussed earlier). At the same time, the philanthropic contribution to research is a relatively untapped resource in India, which is something we will be actively engaged in near future.

## If you could go back in time to when you launched your career, what advice would you give to yourself?

Probably nothing, largely because I know how younger me was averse to any advice.

## Finally, where do you see yourself in 20 years’ time?

It is daunting to think 20 years ahead, so I am going to reduce it to 10 years, which is reasonable. Most likely still in an academic setting, with some industry tie-ups or still trying! One thing I hoped to have achieved is training a handful of people to carry the science baton into the 21st century; anything more than that is a bonus.

